# Context-dependent value of HLA class I diversity in advanced non-small cell lung cancer treated with immune checkpoint blockade

**DOI:** 10.3389/fimmu.2026.1924434

**Published:** 2026-09-29

**Authors:** Javier Ramos-Paradas, Nuria Carrizo, Eva Álvarez, Cristina Martín-Arriscado, Mar López, Nieves Ortiz, Mercedes Herrera, Macarena Rey, Javier Baena, Jon Zugazagoitia, Luis Paz-Ares, Álvaro C. Ucero

**Affiliations:** 1 Cancer Area, Health Research Institute Hospital 12 de Octubre (lmas 12), Madrid, Spain; 2 H120-CNIO Lung Cancer Clinical Research Unit, Spanish National Cancer Research Center (CNIO), Madrid, Spain; 3 Department of Pathology, Yale University School of Medicine, New Haven, CT, United States; 4 Medical Oncology Department, 12 de Octubre Hospital, Madrid, Spain; 5 Department of Tumors of the Respiratory Tract, Spanish Center for Biomedical Research Network in Oncology (CIBERONC), Madrid, Spain; 6 Physiology and Medicine Departments, Translational Research in Precision Oncology Group, School of Medicine, Complutense University of Madrid, Madrid, Spain

**Keywords:** chemo-immunotherapy, HLA class I diversity, immune checkpoint inhibitors, non-small cell lung cancer, PD-L1, predictive biomarkers, progression-free survival, tumor mutational burden

## Abstract

**Background:**

Immune checkpoint inhibitors (ICI), alone or in combination with chemotherapy, are the standard first-line treatment for advanced non–small cell lung cancer (NSCLC) without actionable oncogenic drivers. Programmed death-ligand 1 (PD-L1) expression and tumor mutational burden (TMB) are routinely used biomarkers, yet both show limited predictive accuracy at the individual level, particularly in the context of chemo-immunotherapy. HLA class I diversity has been proposed as a biomarker of response to ICI monotherapy, although its role in first-line chemo-immunotherapy remains unclear.

**Methods:**

We performed a retrospective validation and exploratory study including patients with stage IV NSCLC treated with ICI monotherapy or platinum-based chemo-immunotherapy in the first-line setting. Tumors were profiled using a single clinically-validated targeted sequencing assay (TruSight Oncology 500), allowing simultaneous assessment of TMB and HLA class I diversity. Furthermore, tumors had a complete clinical annotation, including the level of PD-L1 expression. Associations with objective response rate (ORR) and progression-free survival (PFS) were evaluated.

**Results:**

PD-L1 expression and TMB showed limited discriminative performance for ORR, particularly in chemo-immunotherapy–treated patients. Composite HLA class I diversity did not predict ORR or PFS. A triple-negative biomarker profile identified a subgroup with reduced likelihood of response, although statistical significance was not reached. Exploratory analyses suggested that locus-specific HLA-B diversity, when combined with TMB, was associated with numerically longer PFS, whereas HLA-A, HLA-C, and composite HLA metrics showed no consistent association.

**Conclusions:**

In advanced NSCLC treated predominantly with first line chemo-immunotherapy, composite HLA class I diversity does not improve outcome prediction beyond established biomarkers. Locus-specific HLA metrics, particularly HLA-B, may warrant further investigation.

## 1 Introduction

Lung cancer remains the leading cause of cancer-related mortality worldwide, accounting for approximately 1.8 million deaths annually. Non–small cell lung cancer (NSCLC) represents nearly 85% of all lung cancer cases and is frequently diagnosed at advanced stages, when curative treatment is no longer feasible and systemic therapy constitutes the mainstay of management (1–4). Despite major therapeutic advances over the last decade, long-term outcomes for patients with metastatic NSCLC remain poor, underscoring the need for improved treatment stratification (5).

In the era of precision oncology, multiple parameters are evaluated to optimize NSCLC management, including histology, stage, presence of metastasis, clinical characteristics and tumor programmed death-ligand 1 (PD-L1) expression, among others. One of the current cornerstones of NSCLC treatment decision consists of developing a comprehensive molecular profile based on next generation sequencing to identify genomic alterations that could be therapeutically exploited with available targeted therapies (6, 7). Given this complex context, a broad overview of first-line care of advanced NSCLC comprises the use of targeted therapies if actionable oncogenic drivers are present, while if absent, immune checkpoint inhibitors (ICI) against the PD-1/PD-L1 axis become the therapy of choice, guided by tumor PD-L1 expression. ICI monotherapy is recommended for patients with high PD-L1 expression, most commonly defined as a tumor proportion score (TPS) ≥50%, whereas platinum-based chemotherapy combined with anti–PD-(L)1 therapy has become the standard of care for the majority of patients with lower PD-L1 expression (4, 8–12). Consequently, chemo-immunotherapy currently represents the most frequently used first-line strategy worldwide. However, even within biomarker-enriched populations, only a subset of patients achieves durable clinical benefit, highlighting the limitations of existing predictive markers (5, 13–16).

In this context, there is significant interest in improving the prediction of response to immunotherapy. Some research is focused on expanding the knowledge of well-established predictive biomarkers, such as PD-L1 and tumor mutational burden (TMB). Other studies are exploring the potential predictive capacity of putative markers, such as HLA-I diversity, mutation signatures, genetic polymorphisms, distribution and phenotype of Tumor Infiltrating Lymphocytes (TILs) and other immune cells, Tertiary Lymphoid Structures (TLS), transcriptomic and epigenetic profiles, gut microbiome or blood-derived markers such as ctDNA, among others. Recently, the combination of multiple biomarkers to improve their individual predictive performance is an additional strategy (17–20).

PD-L1 expression and TMB are the most widely used biomarkers to guide ICI-based therapies. Multiple studies have demonstrated that a higher PD-L1 expression and TMB are consistently associated with a better ratio and duration of response to PD(L)1 inhibitors (4, 21–26). Altogether, these two biomarkers have substantially improved the clinical benefit obtained from immunotherapy and redefined the paradigm of advanced NSCLC management, predominantly at the population level. However, their predictive accuracy at the individual level is limited, particularly in the context of chemo-immunotherapy, where clinical benefit extends across broad biomarker-defined subgroups (25, 27). This has motivated the search for additional biomarkers capable of capturing complementary aspects of tumor–immune interactions beyond PD-L1 expression and neoantigen load.

Human leukocyte antigen (HLA) class I molecules play a central role in antigen presentation and CD8^+^ T-cell–mediated immune recognition, providing a mechanistic link between tumor genomics and immune surveillance (28, 29). HLA class I genotype and diversity influence the breadth of peptide presentation and have been associated with response to immune checkpoint blockade across multiple tumor types (30, 31). Several studies have proposed quantitative metrics summarizing HLA class I diversity, including evolutionary divergence–based approaches, and have reported associations with response to PD-(L)1 blockade (PD-1/PD-L1 axis blockade). More recently, HLA-related metrics have also been evaluated in patients receiving PD-(L)1 blockade combined with chemotherapy, raising the possibility that the predictive value of HLA-I diversity may differ in the chemo-immunotherapy setting (32). Because several cytotoxic agents can induce immunogenic cell death, increasing tumor antigen release and reshaping the tumor immune microenvironment (33–35), whether HLA-I diversity retains predictive value in the chemo-immunotherapy setting remains largely unknown.

Cuppens and colleagues systematically evaluated HLA class I diversity derived from targeted sequencing in patients with advanced NSCLC treated with immune checkpoint inhibitors as monotherapy, and proposed a categorical definition of high HLA diversity based on upper-percentile diversity at any HLA-A, -B, or -C locus (36). Using this composite definition, they reported associations with clinical benefit in selected analyses, although results varied depending on the clinical endpoint assessed. Importantly, the applicability of this HLA diversity framework to first-line chemo-immunotherapy—now the dominant treatment paradigm in advanced NSCLC—has not been firmly established.

In this retrospective validation and exploratory study, we analyzed two clinically annotated cohorts of patients with advanced NSCLC treated in the first-line setting, either with immunotherapy in monotherapy or chemo-immunotherapy, and profiled using a single clinically validated targeted sequencing assay. Our objectives were to (i) assess the performance of established biomarkers, including PD-L1 and TMB, in predicting treatment response; (ii) validate the previously proposed HLA class I diversity categorization by Cuppens et al. in the chemo-immunotherapy setting; and (iii) explore whether alternative HLA diversity features provide incremental information for objective response and progression-free survival beyond conventional biomarkers.

## 2 Methods

### 2.1 Study design and patient cohorts

This retrospective observational study was conducted at Hospital Universitario 12 de Octubre (Madrid, Spain). Patients with histologically confirmed advanced NSCLC (most of the patients as stage IV) who received first-line systemic therapy between 2010 and 2025 were eligible for inclusion. All patients had molecular profiling data generated using a targeted next-generation sequencing assay and sufficient clinical follow-up data for response and survival analyses.

Patients were stratified into two cohorts according to standard of care first-line treatment selection, which was mainly guided by PD-L1 expression and the absence of actionable oncogenic drivers, two established-population level biomarkers. Cohort I included patients with tumors exhibiting high PD-L1 expression (TPS ≥50%) who were treated with immune checkpoint inhibitor monotherapy. Cohort II included patients with tumors exhibiting PD-L1 TPS <50% who were treated with platinum-based chemotherapy combined with anti–PD-(L)1 therapy. The specific treatment regime of every patient is shown in **Supplementary Table 1**. Patients with known actionable oncogenic drivers treated with targeted therapies in the first-line setting were excluded. Baseline clinicopathologic characteristics of the study population are summarized in **Table 1**.

**Table 1:** Baseline clinical and molecular characteristics.

Characteristic	Overall (N = 91)	Cohort I: ICI monotherapy (N = 26)	Cohort II: chemo-immunotherapy (N = 65)
Clinical characteristics			
Age at advanced/recurrent diagnosis, years, median [IQR]	64.0 [55.4–72.0]	70.3 [64.0–74.8]	60.7 [53.6–68.9]
Age at first dose, years, median [IQR]	63.9 [55.3–72.6]	69.3 [63.5–74.9]	60.7 [53.7–68.7]
Sex, n (%)			
Male	59 (64.8%)	21 (80.8%)	38 (58.5%)
Female	27 (29.7%)	5 (19.2%)	22 (33.8%)
Unknown/missing	5 (5.5%)	0 (0.0%)	5 (7.7%)
Smoking history, n (%)			
Never smoker	4 (4.4%)	2 (7.7%)	2 (3.1%)
Former smoker	53 (58.2%)	16 (61.5%)	37 (56.9%)
Current smoker	27 (29.7%)	8 (30.8%)	19 (29.2%)
Unknown/missing	7 (7.7%)	0 (0.0%)	7 (10.8%)
ECOG performance status, n (%)			
ECOG 0	23 (25.3%)	5 (19.2%)	18 (27.7%)
ECOG 1	47 (51.6%)	13 (50.0%)	34 (52.3%)
ECOG 2	9 (9.9%)	6 (23.1%)	3 (4.6%)
ECOG 3	3 (3.3%)	2 (7.7%)	1 (1.5%)
Unknown/missing	9 (9.9%)	0 (0.0%)	9 (13.8%)
Histology, n (%)			
Adenocarcinoma	59 (64.8%)	18 (69.2%)	41 (63.1%)
Squamous cell carcinoma	15 (16.5%)	5 (19.2%)	10 (15.4%)
Other	13 (14.3%)	3 (11.5%)	10 (15.4%)
Unknown/missing	4 (4.4%)	0 (0.0%)	4 (6.2%)
Stage at start of PD-1 axis blockade, n (%)			
Stage III	7 (7.7%)	2 (7.7%)	5 (7.7%)
Stage IV	80 (87.9%)	24 (92.3%)	56 (86.2%)
Unknown/missing	4 (4.4%)	0 (0.0%)	4 (6.2%)
Molecular biomarkers			
PD-L1 TPS, %, median [IQR]	1.0 [0.0–61.5]	85.0 [76.2–90.0]	0.0 [0.0–2.0]
PD-L1 TPS ≥1%, n (%)	49 (53.8%)	26 (100.0%)	23 (35.4%)
PD-L1 TPS ≥50%, n (%)	26 (28.6%)	26 (100.0%)	0 (0.0%)
TMB, mut/Mb, median [IQR]	8.4 [3.6–15.1]	7.7 [3.7–11.2]	9.6 [3.6–16.7]
TMB ≥7.847 mut/Mb, n (%)	48 (52.7%)	13 (50.0%)	35 (53.8%)
HLA-A diversity score, median [IQR]	11.6 [3.8–15.8]	11.8 [3.1–15.8]	11.6 [3.9–15.5]
HLA-B diversity score, median [IQR]	9.3 [4.1–13.8]	10.9 [2.9–20.2]	9.3 [4.6–12.9]
HLA-C diversity score, median [IQR]	12.4 [4.4–18.9]	15.9 [6.4–18.7]	9.9 [4.1–19.2]
HLA class I high by Cuppens/internal p85, n (%)	40 (44.0%)	12 (46.2%)	28 (43.1%)

Values are n (%) or median [IQR].

Cohort I: PD-L1 TPS ≥50% treated with ICI monotherapy; Cohort II: PD-L1 TPS <50% treated with chemo-immunotherapy.

### 2.2 Molecular profiling and biomarker assessment

All tumor samples underwent molecular profiling using the TruSight Oncology 500 (TSO500) assay (Illumina, San Diego, CA), according to the manufacturer’s instructions and institutional standard operating procedures. TMB was calculated from somatic single-nucleotide variants and small insertions/deletions and expressed as mutations per megabase (mut/Mb), following previously described harmonization principles (37). A panel-specific cutoff of 7.847 mut/Mb, as previously established and validated for TSO500-based analyses, was evaluated (38).

PD-L1 expression was assessed by immunohistochemistry and reported as a tumor proportion score (TPS). For biomarker analyses outside cohort stratification, PD-L1 positivity was defined as TPS ≥1%, consistent with prior clinical and biomarker studies (8).

HLA class I genotyping and diversity metrics were derived from sequencing data using the DRAGEN pipeline (Illumina, San Diego, CA). Locus-specific diversity scores were generated for HLA-A, HLA-B, and HLA-C. For each HLA locus, the two predicted alleles were translated into their corresponding protein sequences and globally aligned using the Needleman–Wunsch algorithm implemented in EMBOSS needleall (gap-open penalty = 10; gap-extension penalty = 0.5) (39). The resulting alignment score was compared with the distribution of alignment scores obtained from all possible allele combinations for that HLA locus. HLA diversity was expressed as 100 × (1 − quantile), such that higher values represent greater sequence divergence between the two alleles. Composite HLA class I diversity was subsequently defined according to the approach proposed by Cuppens et al. To enable validation of previously published approaches, a composite HLA diversity variable was constructed following the definition proposed by Cuppens et al., whereby tumors were classified as “HLA high” if diversity at any HLA-A, -B, or -C locus fell within the upper 15th percentile of the reference distribution (85th percentile cutoff) (36).

### 2.3 Clinical endpoints

Objective response rate (ORR) was defined according to RECIST criteria, with responders classified as patients achieving complete or partial response (CR/PR) and non-responders as those with stable or progressive disease (SD/PD). Progression-free survival (PFS) was defined as the time from treatment initiation to documented disease progression or death from any cause, whichever occurred first. Patients without an event were censored at the date of last follow-up. Overall survival (OS) was defined as the time from diagnosis of stage IV disease to death or last follow-up. Time-to-event analyses were administratively censored at 60 months.

### 2.4 Statistical analysis

Associations between continuous biomarkers and objective response were evaluated using receiver operating characteristic (ROC) curves and area under the curve (AUC) estimates with 95% confidence intervals (CI). Optimal biomarker cut points were derived using Youden’s J statistic and were considered exploratory. Comparisons between correlated ROC curves were performed using DeLong’s test.

Categorical biomarker analyses, including single-biomarker stratifications and combined biomarker definitions (e.g., Cuppens et al. HLA diversity and “triple-negative” biomarker profiles), were evaluated using contingency tables and Fisher’s exact test. Effect sizes were reported as odds ratios (OR) with 95% CI.

Time-to-event outcomes were analyzed using the Kaplan–Meier method, and survival curves were compared using the log-rank (Mantel–Cox) test. Exploratory Cox proportional hazards regression models were used to estimate hazard ratios (HR) and 95% CI for selected biomarker-defined groups and combinations. The proportional hazards assumption was assessed by visual inspection of log-minus-log survival plots.

To explore the stability of survival associations across alternative HLA diversity thresholds, cut-point stability analyses were performed using the SCRIBE (Survival Cut-point Reporting and Integrative Biomarker Evaluation) framework, which systematically evaluates survival associations across a range of percentile-based thresholds (40). Analyses were performed using the publicly available SCRIBE application (https://scribe45.github.io/Website/) following the methodology described by M Lopez de Rodas et al. (AACR Annual Meeting 2026, Abstract 4121). HLA-B diversity was additionally evaluated as a continuous variable using exploratory Cox proportional hazards regression models. Multivariable models were adjusted for TMB and PD-L1 expression, with additional adjustment for treatment cohort in analyses of the overall study population. To investigate potential non-linear associations between HLA-B diversity and PFS, exploratory restricted cubic spline analyses were performed using four knots placed at the 5th, 35th, 65th, and 95th percentiles of the HLA-B diversity distribution, with non-linearity assessed using a joint Wald chi-square test of the non-linear spline terms. These analyses were performed both in the overall study population and separately in Cohort II.

Because analyses beyond the primary validation objective were exploratory, no formal adjustment for multiple comparisons was performed, and these findings should be interpreted as hypothesis-generating. All statistical tests were two-sided and interpreted with emphasis on effect sizes and confidence intervals, given the retrospective design, limited sample size, and exploratory nature of multi-biomarker analyses. Statistical analyses were performed using GraphPad Prism and R.

## 3 Results

### 3.1 Patient cohorts and molecular profiling overview

A total of 91 patients with advanced NSCLC (mostly stage IV. See **Table 1**) treated in the first-line setting were included in the study. All tumor samples were successfully profiled using the TruSight Oncology 500 assay, allowing the simultaneous assessment of tumor mutational burden (TMB), and HLA class I diversity metrics (**Figure 1A**). Clinical attributes, including immunohistochemical assessment of PD-L1 (as tumor proportion score, TPS) and survival, were registered.

**Figure 1:**
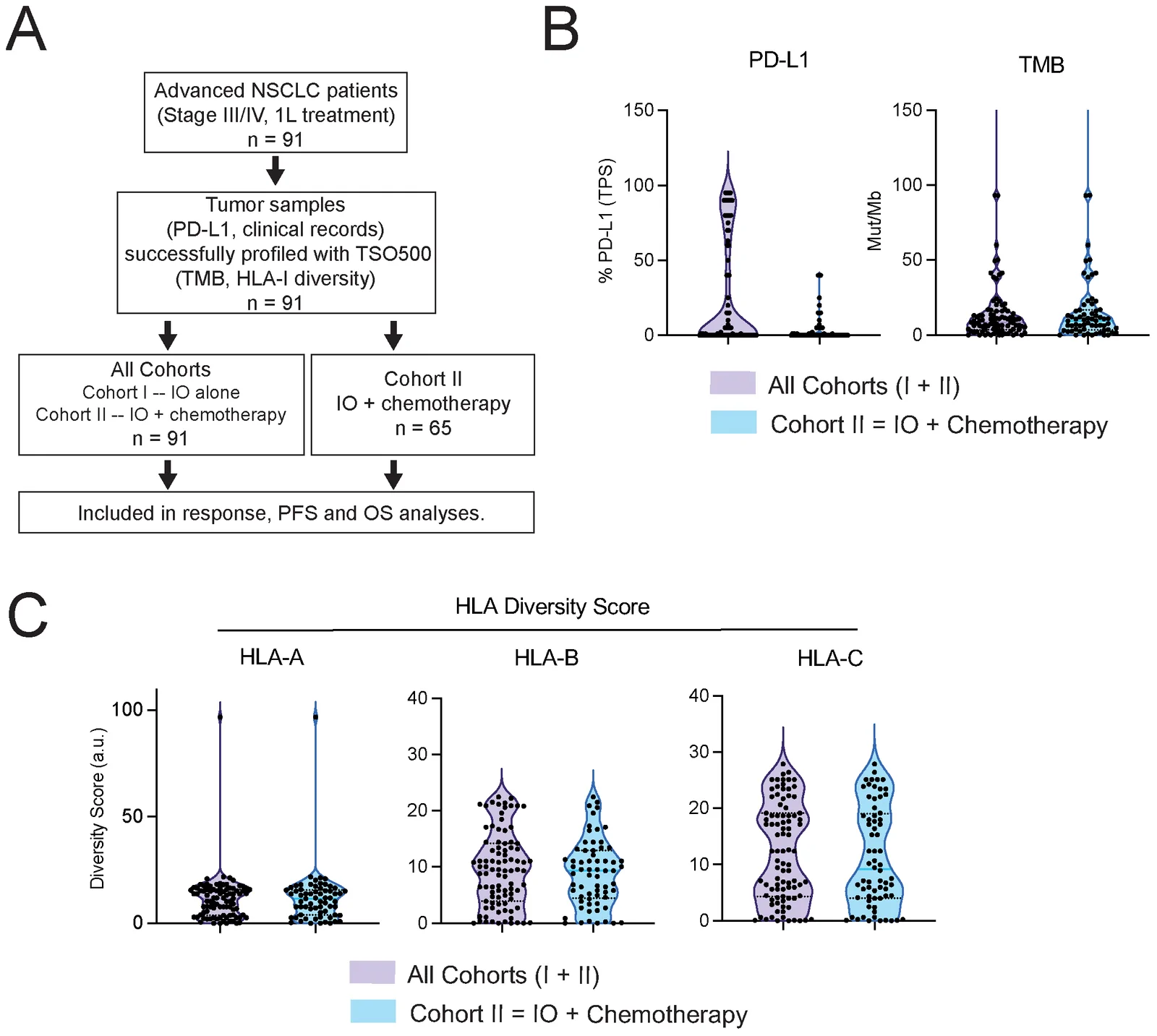
Study design and distribution of molecular biomarkers. **(A)** Flow diagram of patient inclusion and treatment cohorts. **(B)** Distribution of PD-L1 tumor proportion score (TPS) and tumor mutational burden (TMB). **(C)** Distribution of HLA-A, -B and -C diversity scores across treatment cohorts. a.u. = arbitrary units.

Based on standard-of-care first-line treatment selection, patients were stratified into two clinical cohorts: Cohort I, comprising 26 patients with high PD-L1 expression (TPS ≥50%) treated with immune checkpoint inhibitor monotherapy, and Cohort II, comprising 65 patients treated with platinum-based chemotherapy combined with anti–PD-(L)1 therapy (**Figure 1A**). The specific treatment regime of every patient is shown in **Supplementary Table 1**. Given the limited size of Cohort I and the availability of previous larger ICI-monotherapy studies evaluating HLA class I diversity, analyses were conducted in the overall population and separately in Cohort II, which constituted the primary focus of the study. All 91 patients in the analytical cohort were included in response and survival analyses when endpoint data were available.

As expected, PD-L1 expression was higher in “All Cohorts” compared with Cohort II only, influenced by the PD-L1 TPS in Cohort I (≥50%) (**Figure 1B**). TMB values showed a broad distribution in both cohorts, with overlapping ranges (**Figure 1B**). HLA-A, HLA-B, and HLA-C diversity scores also displayed wide inter-patient variability, without clear separation between treatment cohorts (**Figure 1C**).

### 3.2 Limited predictive performance of PD-L1 and TMB for treatment response

We first evaluated the predictive performance of established biomarkers for objective response to treatment. Receiver operating characteristic (ROC) analyses were performed for PD-L1 tumor proportion score (TPS) and TMB using objective response rate (ORR) as the endpoint.

PD-L1 TPS demonstrated limited discriminative ability, with area under the curve (AUC) values close to 0.5 both in all study population as well as in chemo-immunotherapy-treated Cohort II (All cohorts AUC = 0.51; Cohort II AUC = 0.56), indicating near-random classification performance (**Figure 2A**). For TMB, results showed more discrimination capacity with respect to objective response in all conditions analyzed (All cohorts AUC = 0.64; Cohort II AUC = 0.66) although none of the results yielded significant results (**Figure 2B**).

**Figure 2:**
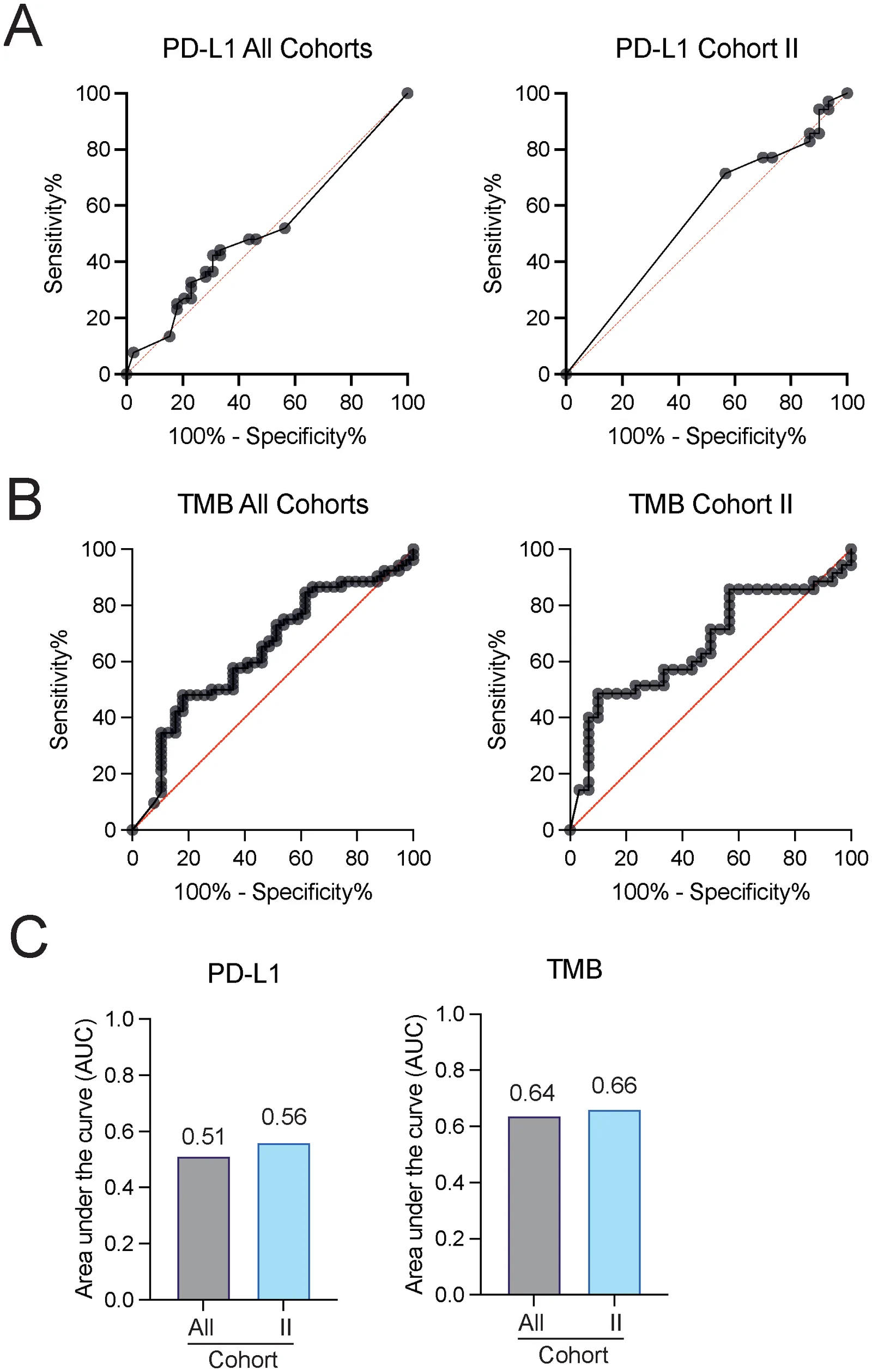
Limited predictive performance of established biomarkers for treatment response. **(A)** ROC curves for PD-L1 TPS in all patients and in patients treated with chemo-immunotherapy. **(B)** ROC curves for tumor mutational burden (TMB) in the overall cohort and in patients treated with chemo-immunotherapy. **(C)** Summary of area under the curve (AUC) values across cohorts.

A direct comparison of AUC values across cohorts confirmed that neither PD-L1 nor TMB provided robust predictive performance for treatment response, either in the overall population or specifically in the chemo-immunotherapy setting (**Figure 2C**). These findings underscore the limited ability of currently used biomarkers to accurately identify responders at the individual-patient level, including those treated with first-line chemo-immunotherapy.

### 3.3 Rationale for extended biomarker analyses

Given the limited predictive performance of PD-L1 and TMB observed in this cohort, we next sought to determine whether HLA class I diversity metrics could provide complementary or incremental information for treatment response. Subsequent analyses therefore focused on (i) validating previously proposed composite HLA diversity definitions and (ii) exploring alternative HLA-based metrics alone and in combination with established biomarkers.

#### 3.3.1 HLA class I diversity does not improve prediction of objective response in first-line-treated NSCLC

We next evaluated whether HLA class I diversity metrics could improve the prediction of objective response rate (ORR), either alone or in combination with tumor mutational burden (TMB), focusing on both the combined study population (Cohorts I+II) and patients treated with chemo-immunotherapy (Cohort II) (**Figure 3**).

**Figure 3:**
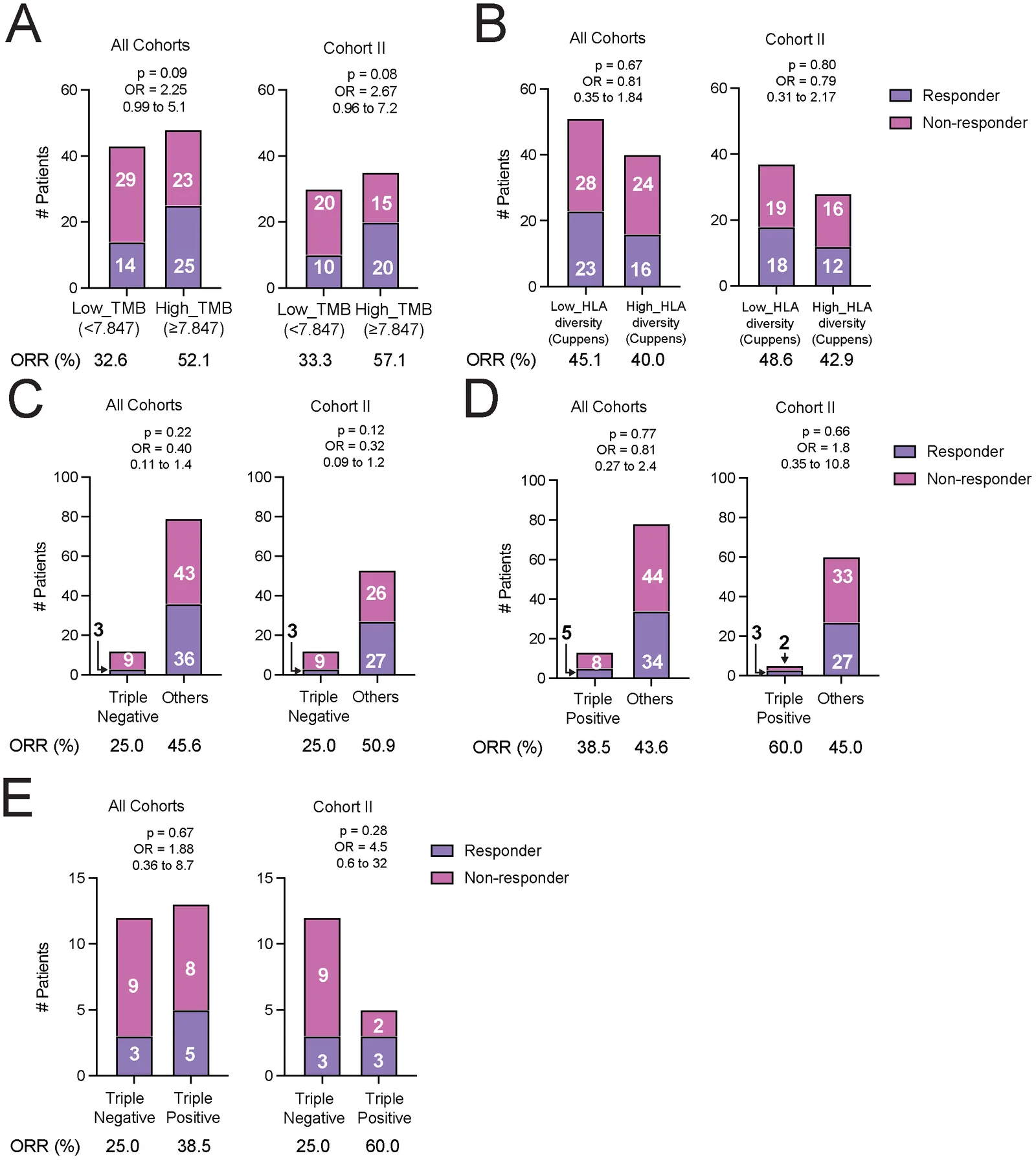
Biomarker performance for objective response rate (ORR) in advanced NSCLC treated with either immunotherapy or chemo-immunotherapy in first line. **(A)** Association between tumor mutational burden (TMB) and objective response in the combined population (Cohorts I+II) and in patients treated with chemo-immunotherapy (Cohort II), using a panel-specific cutoff of 7.847 mut/Mb. **(B)** Association between composite HLA class I diversity defined according to the Cuppens et al. criterion (85th percentile at any HLA-A, -B, or -C locus) and objective response in the combined population and in Cohort II. **(C)** Objective response according to a composite triple-negative biomarker definition (PD-L1 <1%, TMB <7.847 mut/Mb, and low HLA class I diversity by the Cuppens criterion), shown for the combined population and for Cohort II. **(D)** Objective response according to a composite triple-positive biomarker definition (PD-L1 ≥1%, TMB ≥7.847 mut/Mb, and high HLA class I diversity by the Cuppens criterion), shown for the combined population and for Cohort II. **(E)** Objective response comparing triple-positive and triple-negative patients. ORR %, Fisher’s exact test p value, odds ratio and 95% CI are shown in the figure.

Using a panel-specific cutoff of 7.847 mut/Mb (38), high TMB was associated with a numerically higher proportion of responders compared with low TMB in both the combined population and in Cohort II (**Figure 3A**). However, confidence intervals were wide in either analysis (Cohorts I+II: OR = 2.25, 95% CI 0.99–5.10, p = 0.09; Cohort II: OR = 2.67, 95% CI 0.96–7.20, p = 0.08), consistent with a trend toward enrichment of response but limited discriminative power at the individual-patient level.

We then assessed the composite HLA class I diversity definition proposed by Cuppens et al., based on the 85th percentile of diversity at any HLA-A, -B, or -C locus. In contrast to TMB, Cuppens HLA diversity showed no association with objective response in either the combined population or in Cohort II (**Figure 3B**). Response rates were similar between patients with high versus low HLA diversity, with odds ratios close to unity (Cohorts I+II: OR = 0.81, 95% CI 0.35–1.84, p = 0.67; Cohort II: OR = 0.79, 95% CI 0.31–2.17, p = 0.80). To determine whether this lack of association was specific to the composite Cuppens et al. definition or also applied to individual HLA class I loci, we additionally evaluated HLA-A, HLA-B, and HLA-C diversity as separate categorical variables using median-based cutoffs. None of the individual HLA loci showed a consistent association with objective response in either the combined population or the chemo-immunotherapy cohort, indicating that simple locus-specific HLA diversity metrics did not improve response discrimination in this setting (**Supplementary Figure S1**).

Lastly, we evaluated a composite triple-negative biomarker definition, combining PD-L1 negativity (TPS <1%), low TMB (<7.847 mut/Mb), and low HLA class I diversity according to the Cuppens criterion. This triple-negative profile identified a subgroup with a numerically reduced likelihood of objective response in both the combined population and in Cohort II (**Figure 3C**). Although statistical significance was not reached, response rates were consistently lower in triple-negative patients than in the remaining population (Cohorts I+II: OR = 0.40, 95% CI 0.11–1.40, p = 0.22; Cohort II: OR = 0.32, 95% CI 0.09–1.20, p = 0.12). Conversely, a triple-positive profile, defined by PD-L1 positivity (TPS ≥1%), high TMB (≥7.847 mut/Mb), and high HLA class I diversity according to the Cuppens criterion, did not identify a clearly response-enriched subgroup when compared with all other patients (**Figure 3D**). Response rates were similar in the combined population and only numerically higher in Cohort II, with wide confidence intervals reflecting the small number of triple-positive cases (Cohorts I+II: OR = 0.81, 95% CI 0.27–2.40, p = 0.77; Cohort II: OR = 1.80, 95% CI 0.35–10.80, p = 0.66). Direct comparison of triple-positive and triple-negative patients suggested a numerically higher response rate in the triple-positive group, particularly in Cohort II, but this exploratory contrast estimates were imprecise owing to limited sample size (Cohorts I+II: OR = 1.88, 95% CI 0.36–8.70, p = 0.67; Cohort II: OR = 4.50, 95% CI 0.60–32.00, p = 0.28) (**Figure 3E**).

Additional exploratory biomarker combinations incorporating PD-L1 expression, TMB and composite HLA class I diversity did not identify a reproducible response-enriched subgroup beyond the numerical trends observed for TMB and the contrast between triple-positive and triple-negative profiles (**Supplementary Figure S2**).

Collectively, these results indicate that composite HLA class I diversity as defined by Cuppens et al. does not independently discriminate objective response in first-line NSCLC treated with chemo-immunotherapy. Although the triple-negative biomarker profile identifies a clinically unfavorable subgroup, the observed associations are driven primarily by the absence of established biomarkers rather than by HLA diversity alone, supporting the need for further exploration of alternative HLA-based metrics and endpoints.

#### 3.3.2 Exploratory association of locus-specific HLA diversity with progression-free survival in chemo-immunotherapy–treated NSCLC

We next investigated whether HLA class I diversity metrics were associated with progression-free survival (PFS), focusing on patients treated with first-line chemo-immunotherapy (Cohort II). PFS was analyzed using Kaplan–Meier estimates and compared across biomarker-defined groups using the log-rank test, including all the patients with valid PFS data in months (N = 47; 38 events) (**Figure 4**, **Supplementary Figures S3**-**S5**).

**Figure 4:**
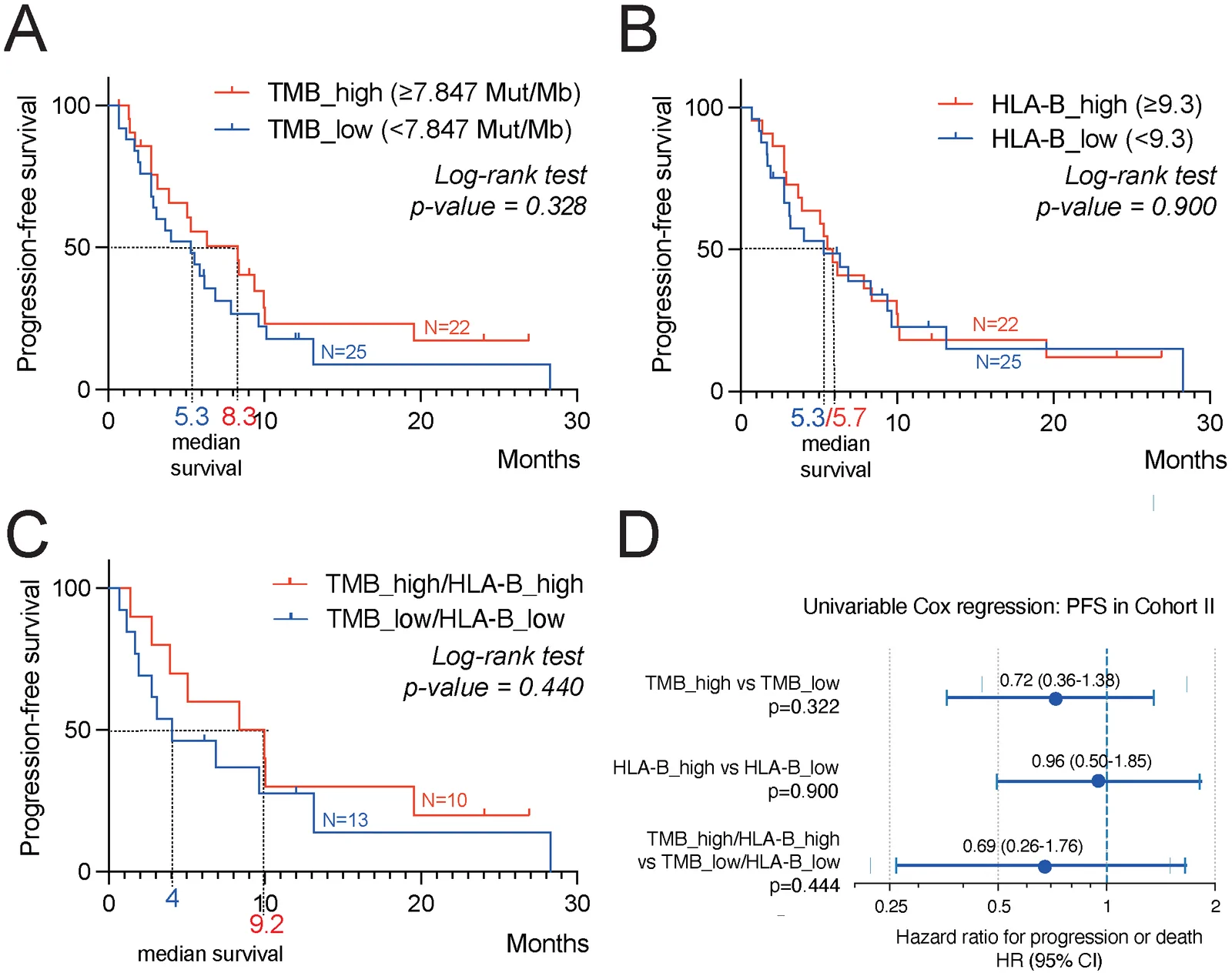
Combined TMB and HLA-B diversity shows a non-significant trend toward improved progression-free survival in Cohort II. Kaplan–Meier analysis of progression-free survival (PFS) in patients from Cohort II according to **(A)** tumor mutational burden (TMB) using the predefined cutoff of 7.847 mut/Mb, **(B)** HLA-B diversity dichotomized by the median split, and **(C)** combined TMB/HLA-B stratification comparing concordant unfavorable patients (TMB_low/HLA-B_low) with concordant favorable patients (TMB_high/HLA-B_high). TMB alone and HLA-B diversity alone did not significantly stratify PFS. The combined TMB_high/HLA-B_high group showed numerically longer median PFS than the TMB_low/HLA-B_low group, although the difference was not statistically significant by log-rank test. **(D)** Forest plot showing hazard ratios (HRs) and 95% confidence intervals from univariable Cox proportional hazards regression for TMB, HLA-B diversity, and the combined TMB/HLA-B classification. P values in panels **A–C** were calculated using the log-rank test; p values in panel D were derived from univariable Cox regression.

When evaluated as single biomarkers, neither tumor mutational burden (TMB) nor locus-specific HLA diversity alone substantially stratified PFS. Using a panel-specific TMB cutoff of 7.847 mut/Mb (38), patients with high versus low TMB showed no statistically significant difference between groups, despite showing different PFS trajectories (median PFS 8.3 vs 5.3 months; log-rank p = 0.328) (**Figure 4A**). Likewise, stratification by HLA-A, HLA-B and HLA-C diversity using a median split did not reveal differences in PFS (HLA-A: median PFS high 5.9 vs low 6.3 months; log-rank p = 0.919; HLA-B: median PFS high 5.7 vs low 5.3 months; log-rank p = 0.900; HLA-C: median PFS high 4.9 vs low 6.9 months; log-rank p = 0.69) (**Figure 4B**, **Supplementary Figure S3**).

Given the absence of significant associations using median-based dichotomization, we next explored whether the prognostic effect of HLA diversity might depend on the cutoff used. SCRIBE analysis across multiple percentile-based thresholds did not identify a consistent association for HLA-A or HLA-C diversity. In contrast, HLA-B showed a more localized pattern at higher percentile thresholds, particularly in Cohort II, where higher HLA-B diversity was associated with lower hazard estimates across a range of upper-percentile cutoffs (**Supplementary Figure S4**). However, this pattern was not supported by continuous-variable analyses. HLA-B diversity was not significantly associated with PFS when modeled as a linear continuous variable, either in the overall study population (adjusted HR 0.97, 95% CI 0.93–1.01; P = 0.153) or in Cohort II (adjusted HR 0.98, 95% CI 0.92–1.04; P = 0.485) (**Supplementary Tables S2**, **S4**). Exploratory restricted cubic spline analyses similarly provided no evidence of a significant non-linear association between HLA-B diversity and PFS, either in the overall population (joint Wald χ^2^ = 3.89, df = 2; P = 0.143) or in Cohort II (joint Wald χ^2^ = 0.19, df = 2; P = 0.911) (**Supplementary Tables S3**, **S5**). Together, these analyses suggest that the HLA-B signal observed in the cut-point analysis is threshold-sensitive and should be considered exploratory.

We next explored whether combining HLA-B diversity with TMB could identify patient subgroups with different outcomes. Patients with both high TMB and high HLA-B diversity showed a numerically longer median PFS than those with both low TMB and low HLA-B diversity (9.2 vs 4.0 months; log-rank P = 0.440), although the difference was not statistically significant (**Figure 4C**). Consistently, Cox regression analysis showed a non-significant reduction in the hazard of progression or death for the TMB-high/HLA-B-high group compared with the TMB-low/HLA-B-low group (HR 0.69, 95% CI 0.26–1.76; P = 0.444) (**Figure 4D**).

Analogous analyses using alternative HLA diversity metrics did not reproduce the exploratory TMB/HLA-B pattern. HLA-A and HLA-C diversity, either alone or in combination with TMB, did not consistently stratify PFS in Cohort II. Similarly, composite HLA class I diversity according to the Cuppens et al. criterion did not identify patients with distinct PFS outcomes, either as a single biomarker or when combined with TMB (**Supplementary Figure S6**). We also evaluated PD-L1-based survival stratifications and additional composite biomarker profiles. PD-L1 expression alone did not clearly discriminate PFS in chemo-immunotherapy–treated patients, and combinations incorporating PD-L1, TMB, HLA-B diversity, did not reveal a consistent survival association exceeding the exploratory signal observed for combined TMB/HLA-B diversity (**Supplementary Figure S7**). Finally, we explored composite biomarker profiles integrating PD-L1 expression, TMB, and the Cuppens HLA diversity classification. Triple-negative patients showed numerically shorter PFS than the remaining patients (median PFS 5.5 vs 6.2 months), whereas triple-positive patients showed numerically longer PFS (10.0 vs 5.5 months). Direct comparison of the two extreme profiles similarly favored the triple-positive group (median PFS 10.0 vs 5.5 months); however, none of these exploratory comparisons reached statistical significance (**Supplementary Figure S8**), although analyses were limited by the small number of patients within the resulting subgroups.

Together, these findings indicate that composite HLA diversity and PD-L1-based combined profiles do not robustly stratify survival outcomes in this setting. In contrast, the combined TMB/HLA-B analysis derived in a hypothesis-generating signal, suggesting that locus-specific HLA-B diversity may warrant further investigation as a potential modifier of TMB-associated benefit in first-line chemo-immunotherapy–treated NSCLC.

## 4 Discussion

In this retrospective validation and exploratory study, we systematically evaluated the performance of HLA class I diversity metrics derived from targeted sequencing as potential biomarkers of response and survival in first-line treated advanced NSCLC. Our analyses yield three main findings. First, established biomarkers such as PD-L1 expression and TMB showed limited predictive performance for objective response in this cohort, including patients treated with chemo-immunotherapy. Second, the composite HLA class I diversity definition proposed by Cuppens et al. did not discriminate objective response or progression-free survival in the chemo-immunotherapy setting. Third, an exploratory, locus-specific association emerged between HLA-B diversity and progression-free survival when combined with TMB, suggesting that not all HLA class I loci may contribute equally to treatment outcomes in this context.

The central aim of this study was to assess the generalizability of the HLA diversity framework proposed by Cuppens et al. (2022) to a contemporary first-line treatment landscape dominated by chemo-immunotherapy. Using the same conceptual definition of high HLA diversity—based on the upper percentile (85^th^) of diversity at any HLA-A, -B, or -C locus—and the same clinical endpoint of objective response, we were unable to validate a meaningful association between composite HLA diversity and treatment response. This lack of validation was consistent across the combined population and the chemo-immunotherapy cohort, as well as across sensitivity analyses exploring alternative cutoffs and biomarker combinations. These findings suggest that the predictive value of composite HLA diversity metrics may be context-dependent and may not readily translate from immune checkpoint inhibitor monotherapy to chemo-immunotherapy regimens.

Several factors may explain these differences. Cuppens et al. primarily evaluated patients treated with immune checkpoint inhibitor monotherapy, a setting in which endogenous tumor antigen presentation and T-cell priming likely play a dominant role in mediating response (36, 41–46). In contrast, chemotherapy exerts pleiotropic immunomodulatory effects, including induction of immunogenic cell death, increased tumor antigen release, and modulation of the tumor microenvironment (47, 48). These effects may partially compensate for, or obscure, differences in antigen presentation capacity linked to global HLA diversity. In this context, composite measures of HLA diversity may be less informative for early radiographic response, as reflected by ORR, than in immune checkpoint inhibitor monotherapy. Our findings align with reports from larger pan-cancer analyses showing that the predictive value of global HLA diversity or evolutionary divergence varies substantially across tumor types and treatment regimens (49).

While composite HLA diversity did not stratify response or survival, additional exploratory analyses provided two relevant observations. First, a triple-negative profile combining PD-L1 negativity, low TMB, and low composite HLA class I diversity identified a subgroup with reduced likelihood of objective response, although effect estimates were small and imprecise. Second, a potential locus-specific signal involving HLA-B diversity emerged in relation to progression-free survival when combined with TMB. Cut-point stability analysis using SCRIBE further showed a localized pattern of lower hazard estimates at higher HLA-B diversity thresholds in the overall population, with a similar but non-significant pattern across corresponding thresholds in Cohort II, whereas no comparable pattern of consecutive thresholds was observed for HLA-A or HLA-C. However, this pattern was not supported by continuous-variable modeling: HLA-B diversity was not significantly associated with PFS when modeled as a linear continuous variable, and restricted cubic spline analyses provided no evidence of a significant non-linear association, either in the overall population or in Cohort II. These findings therefore suggest that the HLA-B signal is sensitive to the choice of threshold and should be regarded as exploratory rather than evidence of a robust continuous prognostic relationship.

Although the present analyses do not establish an independent prognostic effect of HLA-B diversity, a locus-specific contribution of HLA-B to antitumor immunity is biologically plausible. Among HLA class I loci, HLA-B is the most polymorphic and has been implicated as a dominant contributor to peptide presentation breadth and T-cell repertoire shaping (50). Previous studies have shown that locus-specific HLA characteristics, including evolutionary divergence, can influence response to immune checkpoint blockade (30, 31). In addition, Negrao et al. reported associations between HLA evolutionary divergence and clinical outcomes in patients receiving PD-1–based therapies, including combinations with chemotherapy, supporting the notion that HLA-mediated antigen presentation remains relevant in chemo-immunotherapy settings (51). In our study, combined stratification by TMB and HLA-B diversity showed numerically longer PFS among patients with both biomarkers high than among those with both biomarkers low, although this difference was not statistically significant and should therefore be considered hypothesis-generating. Conceptually, TMB and HLA diversity may capture complementary components of tumor immunogenicity, reflecting the generation and presentation of potential tumor neoantigens, respectively. Shohdy et al. reported locus-specific associations between HLA class I features and clinical benefit from immune checkpoint blockade, supporting further investigation of locus-level HLA metrics rather than relying exclusively on composite diversity measures (49).

It is important to emphasize that the HLA-B–associated signal observed in this study should be interpreted as hypothesis-generating. The retrospective design, limited sample size, and multiple exploratory analyses constrain definitive conclusions regarding causality or clinical utility. Furthermore, restricted cubic spline analyses provided no evidence of a significant non-linear association between HLA-B diversity and PFS, either in the overall population or in Cohort II. Accordingly, the exploratory analyses should be interpreted as hypothesis-generating and warrant confirmation in independent prospective cohorts. In particular, the absence of statistically significant p-values and the wide confidence intervals observed in survival analyses indicate limited statistical power. Nevertheless, the localized pattern observed for HLA-B in the cut-point analysis, together with the absence of a comparable pattern for alternative HLA metrics, supports further investigation of this finding in larger, independent cohorts.

From a clinical perspective, our results reinforce the limitations of currently available biomarkers for guiding treatment selection in first-line chemo-immunotherapy–treated NSCLC. While PD-L1 expression and TMB remain useful population-level enrichment tools (22, 52), neither biomarker reliably identifies individual patients who will derive durable benefit (8). Although our findings do not support the immediate clinical use of composite HLA diversity as a predictive biomarker, they illustrate the feasibility of extracting HLA class I diversity from the same clinically-validated targeted sequencing workflow used to assess cancer-associated genomic alterations and TMB. This approach enables the parallel evaluation of tumor-intrinsic genomic features, mutational load, and antigen-presentation-related biomarkers within a single diagnostic platform, supporting the broader integration of immune-genomic information into routine molecular profiling. Our data do suggest that more refined, locus-specific approaches to HLA analysis may hold promise, particularly when integrated with established genomic biomarkers.

This study has several limitations. Its retrospective nature introduces the potential for selection bias and unmeasured confounding. Although multivariable Cox models were adjusted for PD-L1 expression and TMB, recognized markers associate to response to ICI, with additional adjustment for treatment cohort in analyses of the overall study population, more advanced causal inference methods such as propensity score weighting were not applied. Given that the primary objective of this study was biomarker evaluation rather than estimation of treatment effects, and considering the relatively small sample size and limited number of outcome events, conventional multivariable adjustment was considered the most appropriate analytical strategy. The modest cohort size also limits statistical power, particularly for multivariable and interaction analyses, and precludes robust subgroup analyses. Importantly, the limited number of patients treated with immune checkpoint inhibitor monotherapy (Cohort I) prevents a meaningful independent validation of the findings reported by Cuppens et al. in the IO-alone setting. HLA diversity was inferred from short-read targeted sequencing rather than dedicated HLA typing or long-read sequencing technologies. Although this approach reflects real-world clinical implementation and is appropriate for estimating HLA-I diversity in routine clinical practice, long-read sequencing may enable the identification of additional structural variation or highly polymorphic genomic features that could further refine HLA-related biomarkers in future studies (53). It is important to highlight that although the cohorts were classified into general categories according to treatment (ICI monotherapy and chemo-immunotherapy) some heterogeneity existed in the specific treatment regimens received, particularly within the chemo-immunotherapy cohort. Different cytotoxic agents may exert distinct immunological effects, which could influence the relationship between HLA-I diversity and clinical outcome. The prolonged inclusion period (2010–2025) may introduce temporal heterogeneity due to changes in diagnostic and therapeutic standards. Given the modest sample size, treatment-era stratification would result in small and potentially unstable subgroups and was therefore not performed. Residual confounding related to treatment era cannot be excluded and should be addressed in larger contemporary cohorts.

Despite these limitations, this study has several strengths that support the relevance of its findings. The analysis was conducted in a clinically annotated real-world cohort of patients with advanced NSCLC treated in the first-line setting, including a substantial proportion receiving contemporary chemo-immunotherapy. In addition, all tumors were profiled using the same clinically validated next-generation sequencing platform, enabling homogeneous molecular characterization and parallel assessment of tumor genomic alterations, TMB, and HLA class I diversity within a single workflow. Importantly, this study was designed, in part, as a validation of a previously published HLA diversity biomarker framework. Although the results were predominantly negative, such validation efforts are essential to define the clinical boundaries of emerging biomarkers, particularly when moving from immune checkpoint inhibitor monotherapy to chemo-immunotherapy–based treatment strategies.

In conclusion, in this retrospective validation and exploratory analysis of first-line treated advanced NSCLC, composite HLA class I diversity as defined by Cuppens et al. did not improve prediction of objective response or progression-free survival in patients treated with chemo-immunotherapy. An exploratory locus-specific association involving HLA-B diversity and progression-free survival was observed when combined with TMB, suggesting that individual HLA loci may differentially influence treatment outcomes in this context. These findings highlight the complexity of HLA-based biomarker development and underscore the need for larger, prospective studies to clarify the role of locus-specific HLA diversity in shaping response to chemo-immunotherapy.

Our findings also reinforce the concept that no single biomarker is likely to fully capture response to immune checkpoint blockade. Instead, future patient stratification will probably rely on multiparametric models that capture multiple layers of tumor biology, integrating complementary biomarkers spanning tumor genomics, HLA-related features, tumor mutational burden, and comprehensive characterization of the tumor immune microenvironment. Advances in sequencing technologies, including long-read sequencing, together with spatial profiling, liquid biopsy and computational approaches, are expected to facilitate the development of such integrated biomarkers by enabling a more comprehensive assessment of antigen presentation, intratumoral heterogeneity and dynamic tumor–immune interactions (19, 53–57). Within this framework, HLA-I diversity may represent one component of future multiparametric biomarker models rather than a standalone predictor of clinical benefit.

## Data Availability

The datasets presented in this study can be found in online repositories. The names of the repository/repositories and accession number(s) can be found in the article/**Supplementary Material**.
