# Supplementary material for: Context-dependent value of HLA class I diversity in advanced non-small cell lung cancer treated with immune checkpoint blockade
Source: Front Immunol. 2026 Sep 29;17:1924434. doi: 10.3389/fimmu.2026.1924434 (PMC13658205; doi:10.3389/fimmu.2026.1924434)
Supplement: Supplementary file 1 [file DataSheet1.pdf]

Supplementary Material

1.1 Supplementary Figures

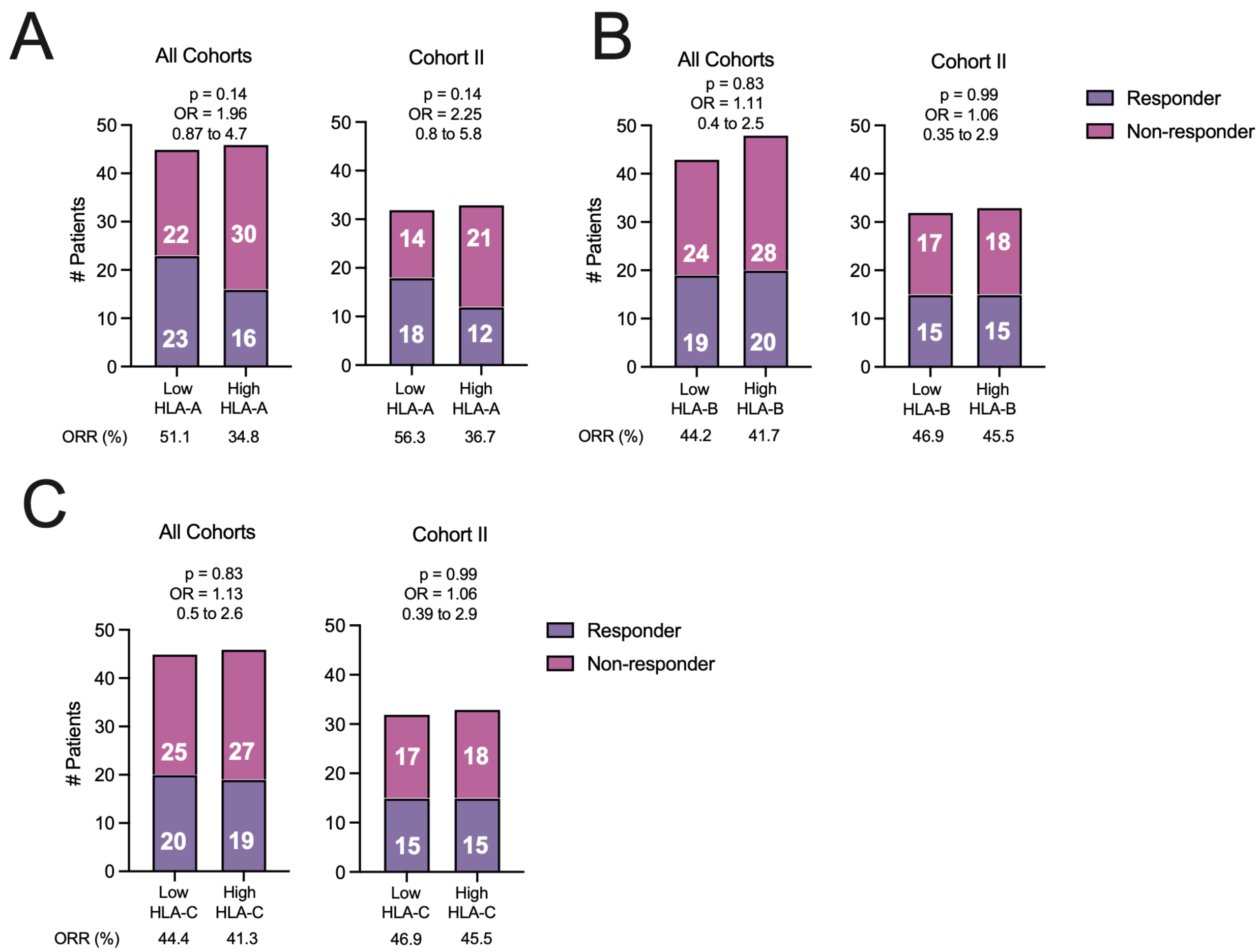

Supplementary Figure S1. Association between locus-specific HLA class I diversity and objective response. Number of responders and non-responders according to locus-specific HLA class I diversity status for HLA-A (A), HLA-B (B), and HLA-C (C). Sample size is expressed on the bars. Analyses are shown for the overall study population, including both treatment cohorts, and for patients treated with first-line chemo-immunotherapy only (Cohort II). HLA-A, HLA-B, and HLA-C diversity were divided as "high" or "low" using median cutoffs. Responders were defined as patients achieving complete or partial response, whereas non-responders included patients with stable or progressive disease according to RECIST criteria. P values were calculated using Fisher's exact test. Odds ratios (ORs) and 95% confidence intervals are shown above each comparison. Objective Response Rate (ORR %) is provided for each condition.

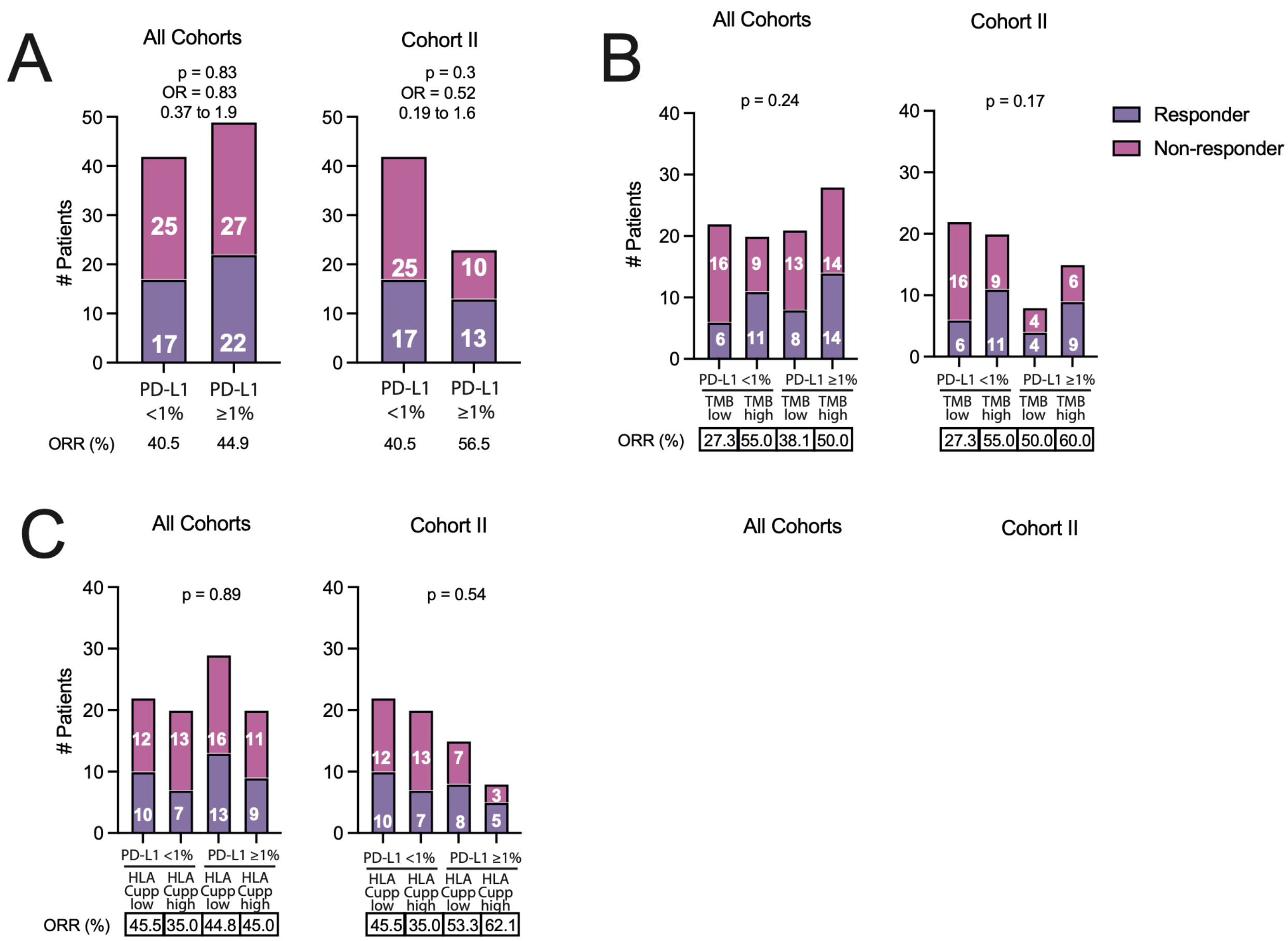

Supplementary Figure S2. Association between combined PD-L1 expression, TMB, HLA class I diversity, and objective response. Number of responders and non-responders according to combined biomarker-defined subgroups. Analyses are shown for the overall study population, including both treatment cohorts, and for patients treated with first-line chemo-immunotherapy only (Cohort II). (A) Objective response according to PD-L1 expression, dichotomized as PD-L1 <1% versus PD-L1 ≥1%. (B) Objective response according to combined PD-L1 expression and tumor mutational burden (TMB) status. (C) Objective response according to combined PD-L1 expression and composite HLA class I diversity defined according to the Cuppens et al. criterion. Responders were defined as patients achieving complete or partial response, whereas non-responders corresponded to patients with stable or progressive disease according to RECIST criteria. Sample size is expressed on the bars. Objective Response Rate (ORR %) is provided for each condition. P values were calculated using the chi-square test.

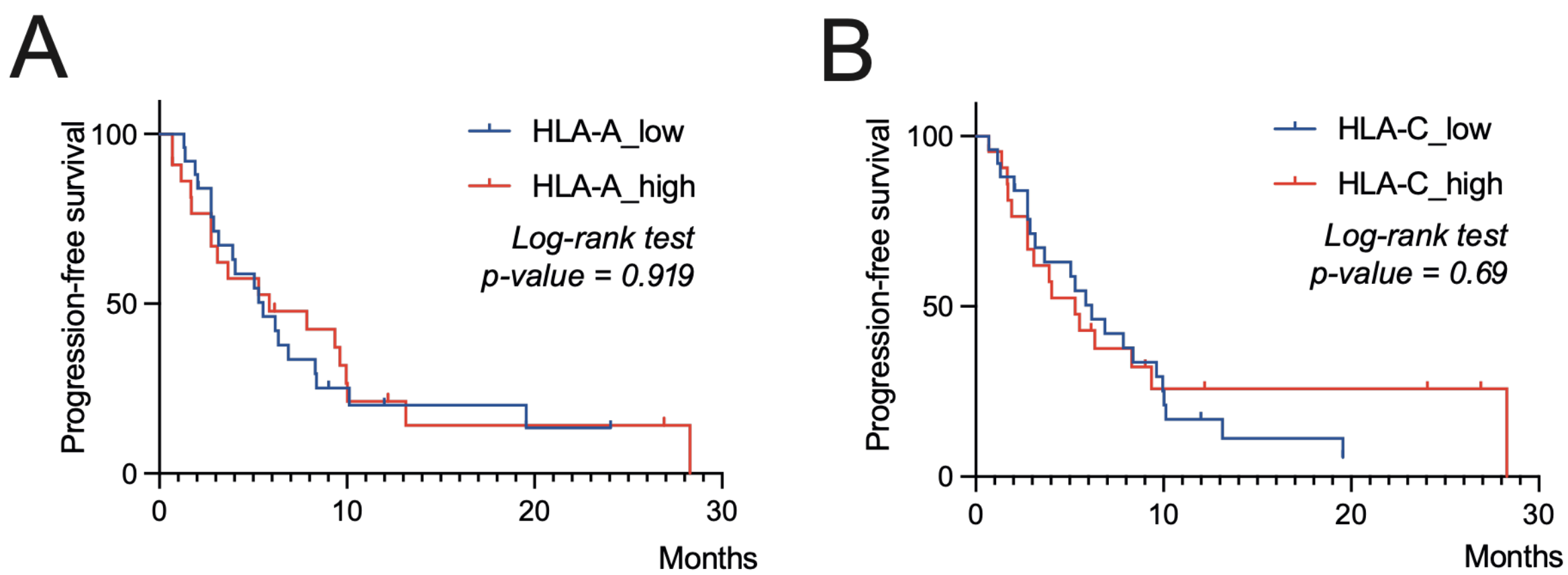

Supplementary Figure S3. Progression-free survival according to locus-specific HLA class I diversity metrics in Cohort II. Progression-free survival according to HLA-A (A) and HLA-C (B) diversity status as individual biomarkers. HLA-A and HLA-C diversity were divided in “high” and “low” using median cutoffs. P values were calculated using the log-rank test.

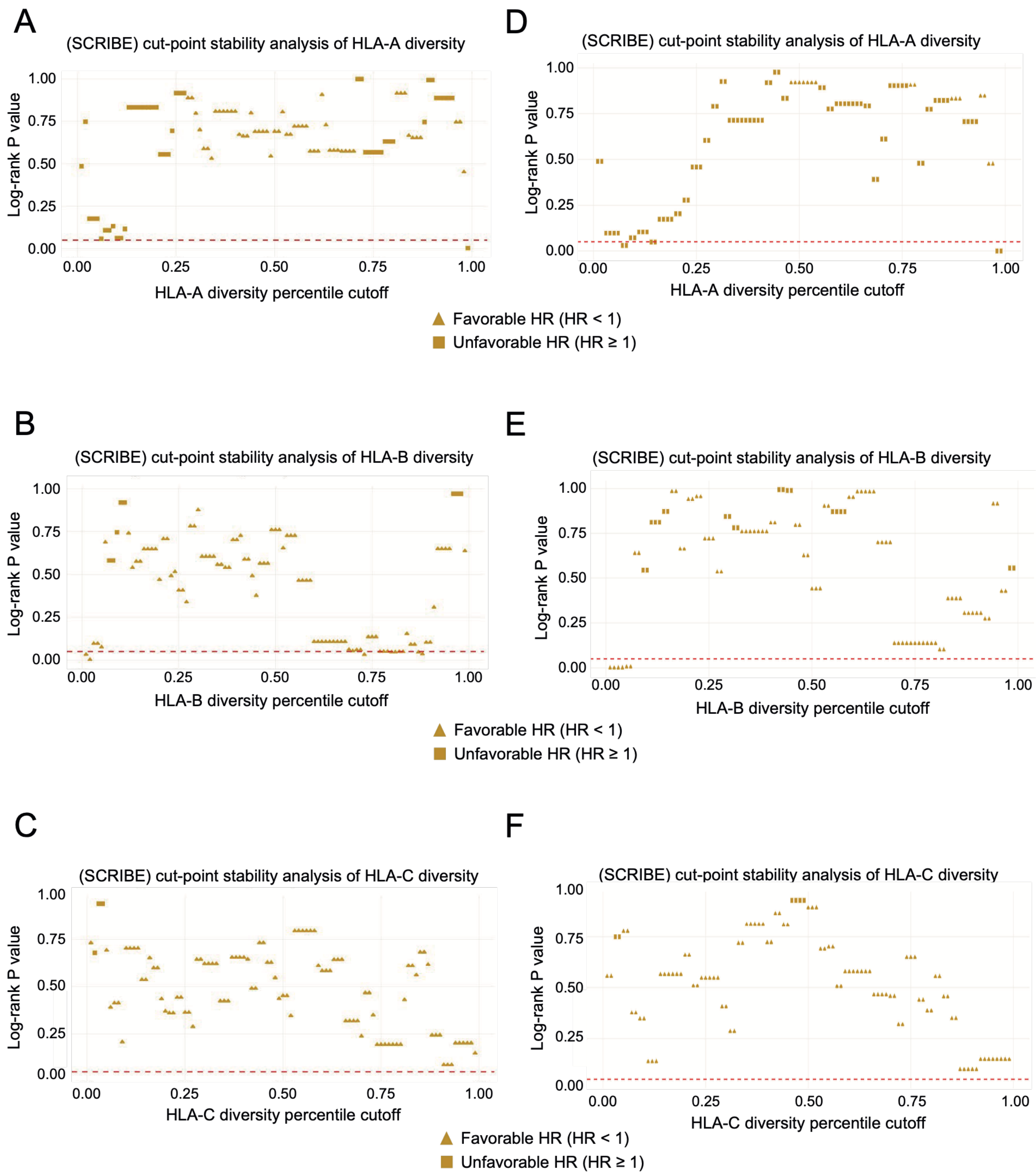

Supplementary Figure S4. Exploratory cut-point stability analysis of HLA class I diversity. Cut-point stability analysis was performed using SCRIBE (see Methods) across candidate percentile thresholds for HLA-A **(A, D)**, HLA-B **(B, E)**, and HLA-C **(C, F)** diversity in the overall population **(A–C)** and Cohort II **(D–F)**. Each symbol represents a different percentile cutoff used to dichotomize HLA diversity. Triangles indicate hazard ratios (HR) <1 and squares HR ≥1. The y-axis shows the log-rank P value for each cutoff, and the dashed horizontal line indicates the nominal significance threshold (P = 0.05).

A

B

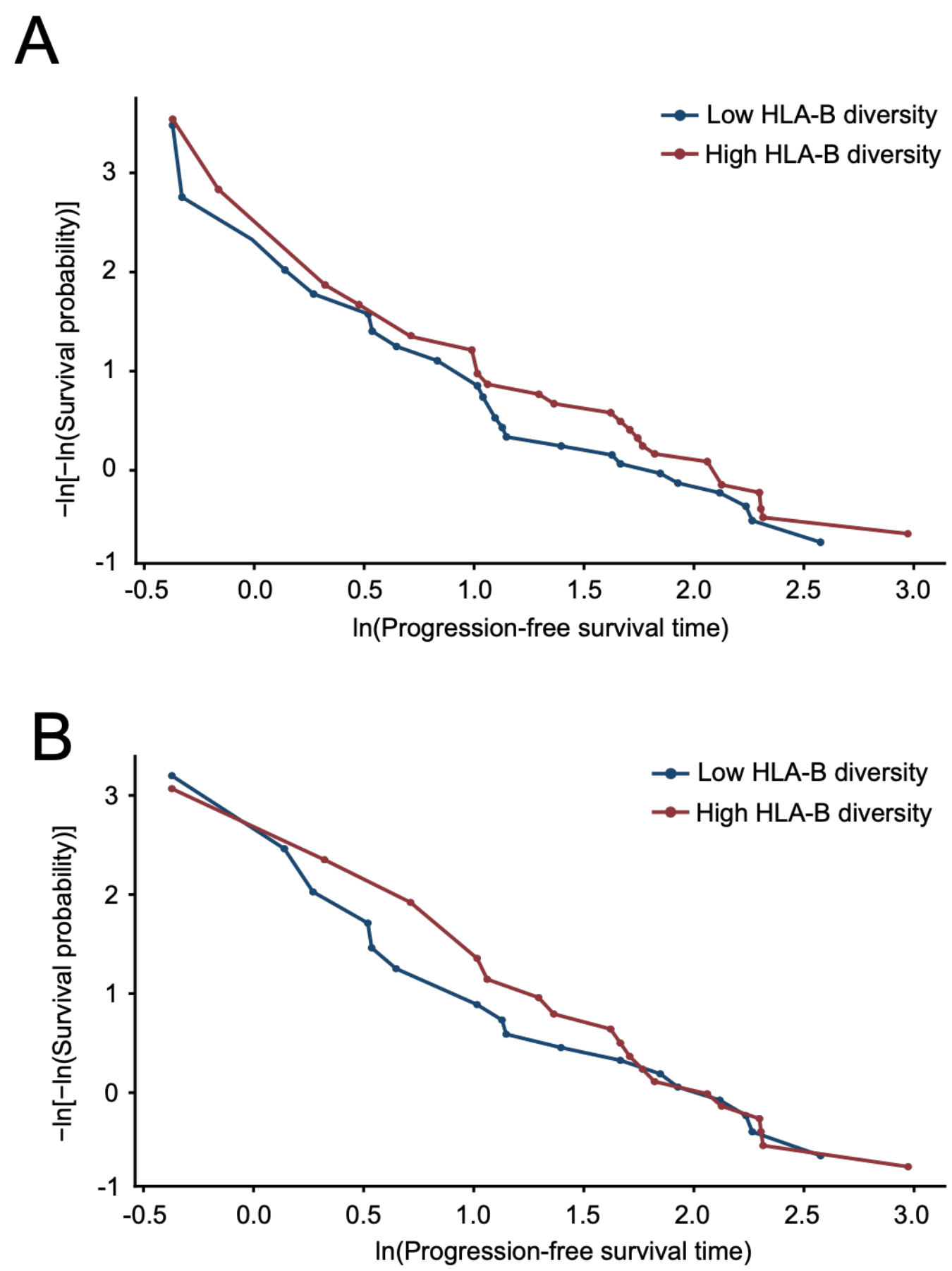

Supplementary Figure S5. Assessment of the proportional hazards assumption using log-minus-log survival plots. (A) Overall study population. (B) Cohort II (chemo-immunotherapy). Visual inspection of the curves did not suggest major violations of the proportional hazards assumption.

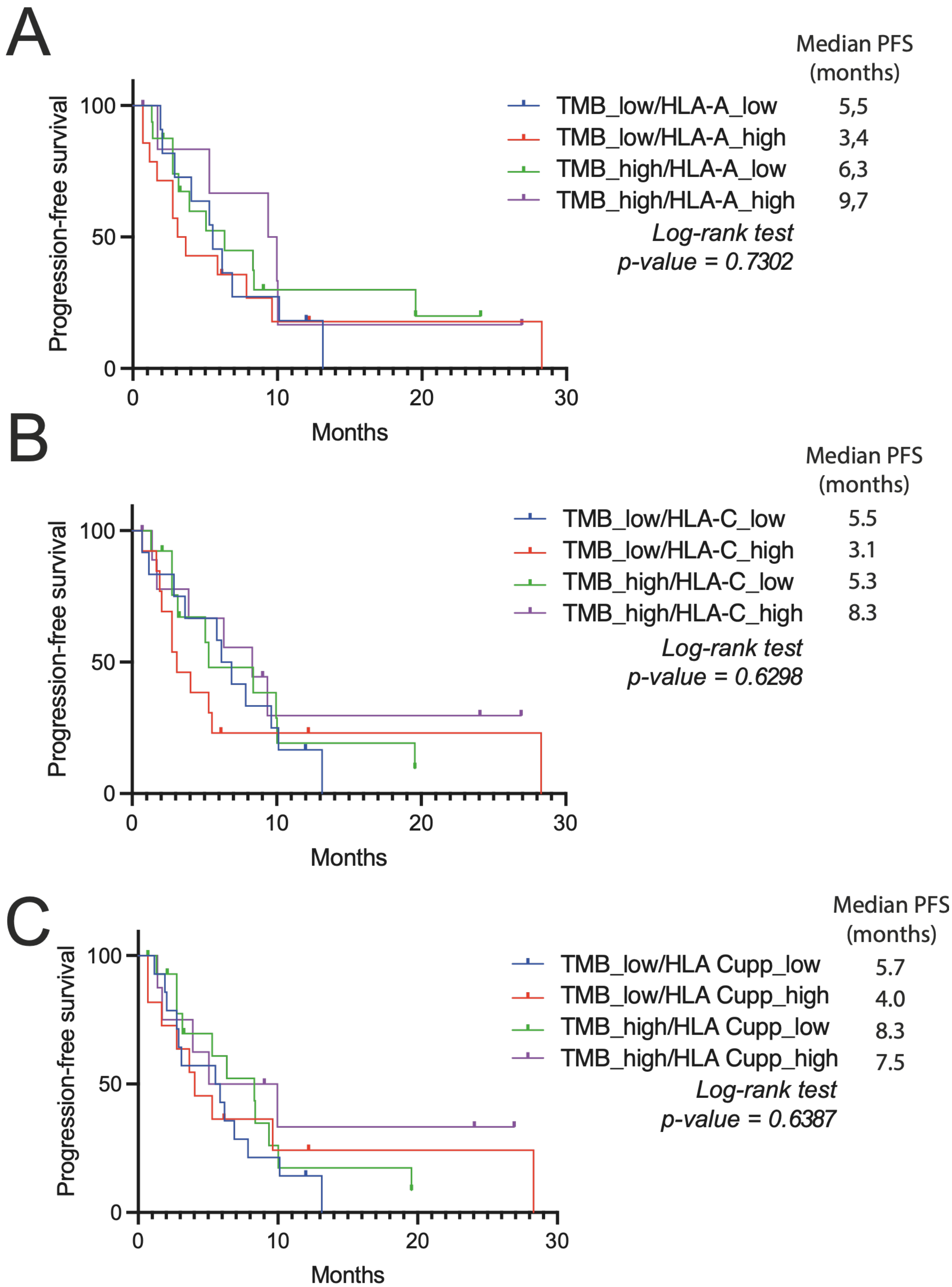

Supplementary Figure S6. Progression-free survival according to combined TMB and alternative HLA class I diversity metrics in Cohort II. Kaplan–Meier curves showing progression-free survival according to combined tumor mutational burden (TMB) status and HLA class I diversity metrics in patients treated with first-line chemo-immunotherapy only (Cohort II). (A)

Progression-free survival according to combined TMB and HLA-A diversity status. (B) Progression-free survival according to combined TMB and HLA-C diversity status. (C) Progression-free survival according to combined TMB and composite HLA class I diversity defined according to the Cuppens et al. criterion.

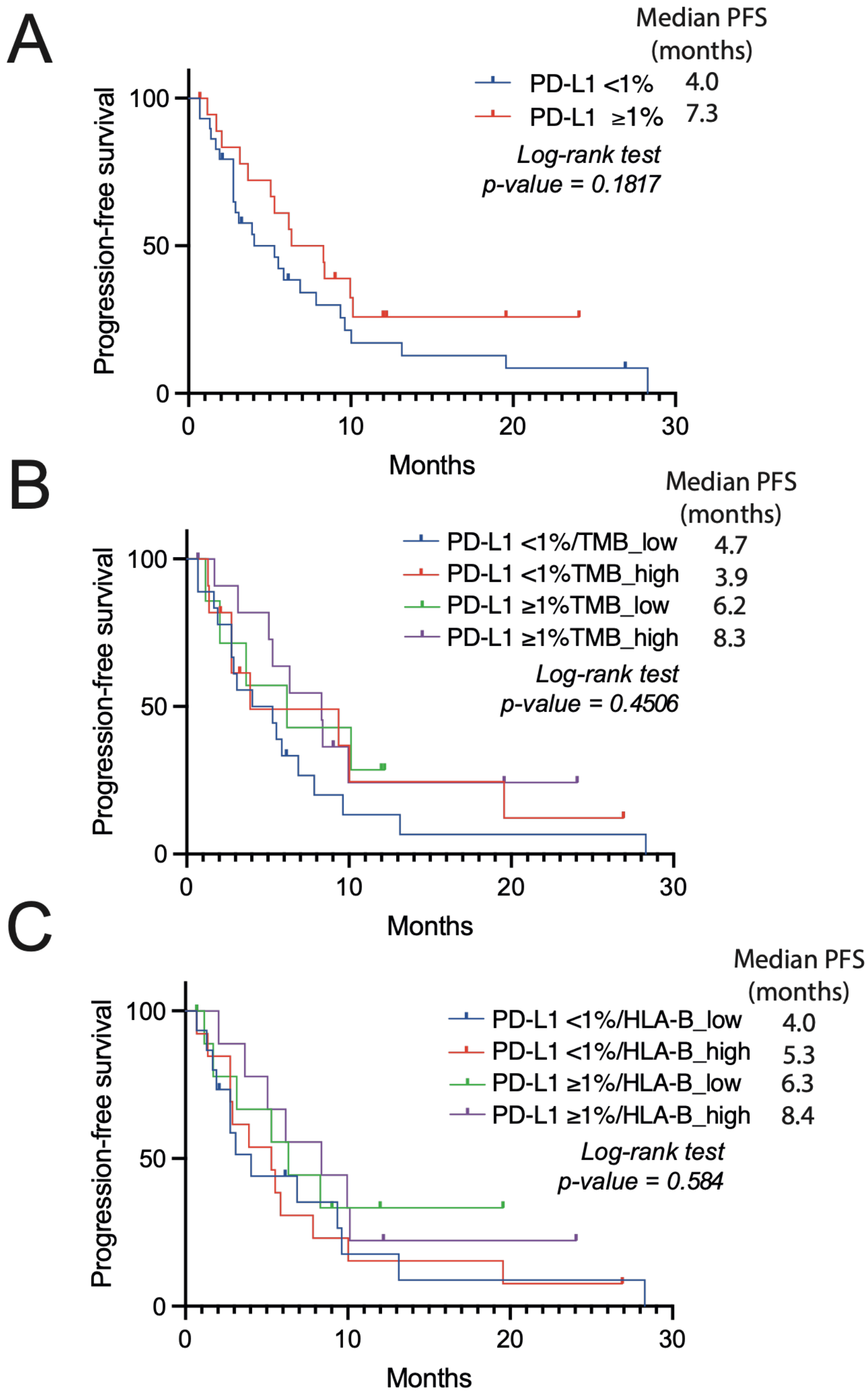

Supplementary Figure S7. Progression-free survival according to PD-L1 expression alone and in combination with TMB or HLA-B diversity in Cohort II. Kaplan–Meier curves showing progression-free survival according to PD-L1 expression and combined biomarker-defined subgroups in patients treated with first-line chemo-immunotherapy only (Cohort II). (A) Progression-free survival according to PD-L1 expression, dichotomized as PD-L1 <1% versus PD-L1 ≥1%. (B) Progression-free survival according to combined PD-L1 expression and tumor mutational burden (TMB) status. (C) Progression-free survival according to combined PD-L1 expression and HLA-B diversity status. Responders and non-responders were not used for these survival analyses; progression-free survival was measured from treatment initiation to disease progression or death.

Median progression-free survival is shown for each group where applicable. P values were calculated using the log-rank test.

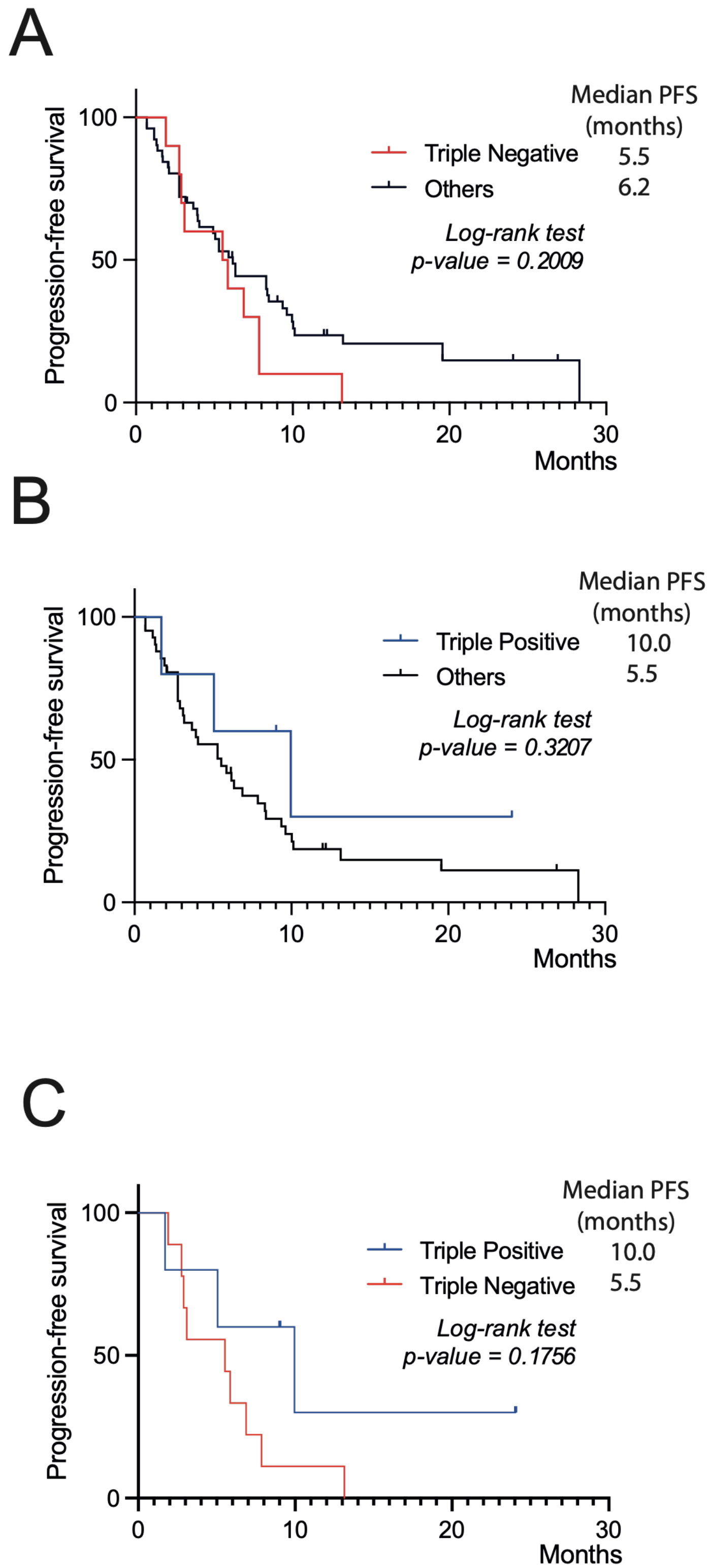

Supplementary Figure S8. Progression-free survival according to triple biomarker combinations in Cohort II. (A) Progression-free survival according to the triple-negative biomarker profile compared with all other patients. The triple-negative group was defined as patients with PD-

L1 <1%, low TMB (<7.847 mut/Mb), and low HLA class I diversity according to the Cuppens et al. criterion. (B) Progression-free survival according to the triple-positive biomarker profile compared with all other patients. The triple-positive group was defined as patients with PD-L1 ≥1%, high TMB (≥7.847 mut/Mb), and high HLA class I diversity according to the Cuppens et al. criterion. (C) Direct comparison of progression-free survival between triple-positive and triple-negative patients. Responders and non-responders were not used for these survival analyses; progression-free survival was measured from treatment initiation to disease progression or death. Median progression-free survival is shown for each group where applicable. P values were calculated using the log-rank test.

Supplementary Table S2. Exploratory Cox proportional hazards analyses evaluating continuous HLA-B diversity and established clinical predictors of progression-free survival

Variable	Univariable HR (95% CI)	P value	Multivariable HR (95% CI)*	P value
**HLA-B diversity (continuous)**	0.97 (0.93–1.01)	0.155	0.97 (0.93–1.01)	0.153†
**Tumor mutational burden (continuous)**	0.99 (0.97–1.01)	0.341	0.99 (0.97–1.01)	0.432
**PD-L1 expression (continuous)**	1.00 (1.00–1.01)	0.201	1.02 (0.99–1.04)	0.129
**PD-L1 positive vs. negative**	0.81 (0.47–1.38)	0.434	0.68 (0.33–1.37)	0.279‡
**Treatment cohort (Qt+IT vs. IT)**	—	—	0.51 (0.23–1.14)	0.102‡

* Multivariable Cox proportional hazards model adjusted for HLA-B diversity, TMB, PD-L1 and treatment cohort.
† Model including continuous PD-L1.
‡ Model including dichotomized PD-L1.
Analyses included patients with evaluable PFS from Supplementary Table S1 (N=68; 55 events).

Supplementary Table S3. Exploratory restricted cubic spline analysis of continuous HLA-B diversity

Variable	HR	95% CI	P value
HLArcs1	0.98	0.74–1.28	0.857
HLArcs2	1.00	1.00–1.00	0.636
HLArcs3	1.00	0.99–1.00	0.450
**Overall test for non-linearity**			
**Test**	**χ^2**	**df**	**P value**
Wald test (HLArcs2 + HLArcs3)	3.89	2	0.143

Restricted cubic spline Cox model adjusted for continuous TMB, dichotomized PD-L1 and treatment cohort. Four knots were placed at the 5th, 35th, 65th and 95th percentiles of the HLA-B diversity distribution in Supplementary Table S1 (0.079, 5.960, 11.045 and 21.162, respectively). HLArcs1–3 are spline basis terms and should not be interpreted as separate clinical predictors. The survival model included N=68 patients with evaluable PFS (55 events).

Supplementary Table S4. Exploratory Cox proportional hazards analyses evaluating continuous HLA-B diversity and established clinical predictors of progression-free survival in Cohort II

Variable	Univariable HR (95% CI)	*P* value	Multivariable HR (95% CI)*	*P* value
**HLA-B diversity (continuous)**	0.98 (0.92–1.04)	0.470	0.98 (0.92–1.04)	0.485†
**Tumor mutational burden (continuous)**	0.99 (0.97–1.01)	0.276	0.99 (0.97–1.01)	0.341†
**PD-L1 expression (continuous)**	1.02 (0.98–1.05)	0.284	1.02 (0.98–1.05)	0.286†
**PD-L1 positive vs. negative**	0.62 (0.32–1.23)	0.170	0.69 (0.34–1.41)	0.307‡

* Multivariable Cox proportional hazards model adjusted for HLA-B diversity, TMB and PD-L1.
† Model including continuous PD-L1.
‡ Model including dichotomized PD-L1.
Analyses included Cohort II patients with evaluable PFS from Supplementary Table S1 (N=47; 38 events).

Supplementary Table S5. Exploratory restricted cubic spline analysis of continuous HLA-B diversity in Cohort II

Variable	HR	95% CI	P value
HLArcs1	0.98	0.71–1.34	0.882
HLArcs2	1.00	1.00–1.00	0.862
HLArcs3	1.00	0.99–1.01	0.806
**Overall test for non-linearity**			
**Test**	**χ^2**	**df**	**P value**
Wald test (HLArcs2 + HLArcs3)	0.19	2	0.911

Restricted cubic spline Cox model adjusted for continuous TMB and dichotomized PD-L1. Four knots were placed at the 5th, 35th, 65th and 95th percentiles of the HLA-B diversity distribution in Cohort II of Supplementary Table S1 (0.071, 5.801, 11.045 and 20.370, respectively). HLArcs1–3 are spline basis terms and should not be interpreted as separate clinical predictors.. The survival model included N=47 patients with evaluable PFS (38 events).
